# 

**Published:** 1907-09

**Authors:** 


### SOUTHERN MEDICAL ASSOCIATION.

The Southern and Gulf Branch of the American Medical Association, comprising the Georgia, Alabama, Tennessee, Mississippi, Louisiana, Florida and Kentucky, will hold a meeting in Birmingham, Ala., September 24-25-26, 1907.

It is hoped that this meeting will be well attended on account of the excellent program now being arranged, and that members of the component State Medical Societies will send at once their application for membership to Raymond Wallace, M. D., Sec'y, Chattanooga, Tenn.

The officers and councilors are:

Henry Hagar Martin, M. D., Prest., Savannah, Ga.; Mack Rogers, M. D., Vice-Prest., Birmingham, Ala.; J. B. Cowan, M. D., Vice-Prest., Tullahoma, Tenn.; J. R. Tackett, M. D., Vice-Prest., Meridian, Miss.; Raymond Wallace, M. D., Secretary, Chattanooga, Tenn.; Y. L. Abernathy, M. D., Treasurer, Hill City, Tenn.

COUNCILORS.—George C. Savage, M. D., Nashville, Tenn.; D. F. Talley, M. D., Birmingham, Ala.; Michael Hoke, M. D., Atlanta, Ga.; John MacDiarmid, M. D., DeLand, Fla.; W. W. Crawford, M. D., Hattiesburg, Miss.; E. D. Martin, M. D., New Orleans, La.

OFFICERS OF SECTIONS—Section of Surgery, F. W. Parham, M. D., Chairman, New Orleans, La.; Jere L. Crook, M. D., Secretary, Jackson, Tenn.; Section on Medicine—Thos. D. Coleman, M. D., Chairman, Augusta, Ga.; J. S. McLester, M. D., Secretary, Birmingham, Ala.; Section on Ophthalmology—L. G. Woodson, M. D., Chairman, Birmingham, Ala.; Oscar Dowling, M. D., Secretary, Shreveport, La.

